# Trends in Organic Food Choices and Consumption: Assessing the Purchasing Behaviour of Consumers in Greece

**DOI:** 10.3390/foods14030362

**Published:** 2025-01-23

**Authors:** Teresa Madureira, Fernando Nunes, José Veiga, Fernando Mata, Maria Alexandraki, Lamprini Dimitriou, Ermioni Meleti, Athanasios Manouras, Eleni Malissiova

**Affiliations:** 1 Centre for Research and Development in Agrifood Systems and Sustainability, Instituto Politécnico de Viana do Castelo (IPVC), 4900-347 Viana do Castelo, Portugal; fnunes@esa.ipvc.pt (F.N.); fernandomata@ipvc.pt (F.M.); 2 Escola Superior Agrária (ESA), Instituto Politécnico de Viana do Castelo (IPVC), 4900-347 Viana do Castelo, Portugal; 3 Escola Superior de Tecnologia e Gestão (ESTG), Instituto Politécnico de Viana do Castelo (IPVC), 4900-347 Viana do Castelo, Portugal; jmcveiga@estg.ipvc.pt; 4 Food of Animal Origin Laboratory, Animal Science Department, University of Thessaly, 41500 Larisa, Greece; alexandraki@uth.gr (M.A.); ldimitriou@uth.gr (L.D.); ermeleti@uth.gr (E.M.); malissiova@uth.gr (E.M.); 5 Nutrition and Dietetics Department, University of Thessaly, 42100 Trikala, Greece; amanouras@uth.gr

**Keywords:** best-worst scaling, Greece, organic food, consumer behaviour

## Abstract

Consumer interest in organic food has surged globally, driven by health, sustainability, and ethical considerations. Key factors include perceived safety, nutritional benefits, and environmental impact, while high prices and limited availability remain barriers. This study examines the factors influencing organic food preferences among Greek consumers, focusing on attribute importance, demographic variations, purchasing locations, and regional differences. A sample of 250 consumers was analysed using a best-worst scaling methodology to research the importance of organic food attributes. The two main attributes were then further analysed using ordinal regression models. Health benefits, particularly the absence of chemicals, emerged as the most valued attribute, followed by nutritional value, absence of GMOs, better taste expectations, and environmental impact. Certification showed intermediary importance, and price, country of origin, availability, and natural appearance were found to have lower importance. Women and highly educated individuals show greater recognition of organic food’s health and nutritional advantages. Consumers in rural regions exhibited stronger preferences for organic products, influenced by cultural traditions, trust in local sourcing, and economic accessibility, while urban consumers displayed more scepticism. For the Greek participants included in the study, supermarkets dominate organic food sales due to affordability and convenience.

## 1. Introduction

In recent years, there has been a significant increase in consumer interest in eating organic food, signalling a remarkable change in dietary preferences and purchasing behaviours [1,2]. Organic foods, defined by strict production standards and regulations aimed at minimising the use of synthetic chemicals, pesticides, fertilisers, genetically modified organisms (GMOs), antibiotics, or irradiation processes [3,4], have gained traction due to their possible promotion of health and nutritional benefits [5,6,7,8], and environmental sustainability [9,10,11,12]. This has attracted the attention of a growing segment of health-conscious and environmentally aware consumers [13,14].

The global organic food market has experienced remarkable growth, due to the increasing awareness of the potential health and nutritional benefits and environmental impact of food choices [15,16]. Consumers are increasingly attracted to organic food because of its safety, nutritional value, nutritional quality, and ethical production practices [3,17,18,19], and also reduced exposure to toxic chemicals [5,20]. Additionally, factors such as taste, freshness, and sensory appeal contribute to the market appeal of organic products [6,21]. Despite the growing popularity of organic foods, consumer attitudes towards them are multifaceted and influenced by various factors [20,22,23]. While some consumers prioritise health and wellness, others are motivated by animal welfare concerns, environmental sustainability, or simply a desire for higher quality and healthier food options [24,25,26].

Consumers have realised in recent decades that their purchasing habits have a direct impact on the environment [27]. The growing consumer’s interest in sustainable food systems regarding production and consumption increases the potential impact of sustainability considerations on consumers’ purchase decisions and emphasises the role of sustainability as a product attribute in consumers’ evaluation of products [17]. Health and food safety is one of the most globally cited factors, which is also the case among Greek consumers, who often associate organic products with health benefits, such as the absence of pesticides and chemical fertilisers, as well as a lower risk of food contamination [28]. The natural aspect of organic products is commonly cited by consumers as a significant driver for their purchase decisions, as it is often associated with the irregular shapes, natural colours, and “imperfect” appearance of organic products with authenticity and minimal processing. This makes them appear closer to their original, farm-fresh state, which many find appealing [29,30,31]. The more natural aspect of organic products is often associated with shorter distribution channels, which many consumers value highly, as products that come from local or regional producers are perceived as fresher, more trustworthy, and environmentally friendly [5,32,33,34].

The origin of organic products plays a crucial role as a cue for quality, and this aligns with the broader consumer perception of organic foods. Emphasising the origin of organic products aligns with consumer values of naturalness, sustainability, and ethical consumption. This makes the origin not just a label but a critical quality cue in purchasing decisions [20,35,36].

GMO ingredients in food are often perceived as risky by consumers, largely due to a lack of understanding about their presence in food and potential health or environmental impacts. Organic products provide reassurance to consumers because organic certification processes explicitly regulate the use of GMOs, ensuring they are absent or present only in minimal amounts due to accidental contamination. This gives consumers confidence in the safety and natural composition of organic foods [37].

Scepticism about the authenticity of organic products can pose a barrier, particularly for less frequent or less informed consumers. In this context, certification labels play a crucial role in building trust and encouraging purchases [38,39,40,41,42]. Recognisable certification logos, such as the EU Organic logo or local equivalents, are key tools for instilling confidence, especially in mainstream supermarkets where consumers may not interact directly with producers. Consumers who buy organic products occasionally—often in generic supermarkets—rely heavily on labels because they lack the deep knowledge or established trust that regular buyers may have. Effective labelling can help bridge this gap and attract these occasional buyers to repeat purchases [43,44,45].

In Greece, organic food consumption remains relatively low but is experiencing steady growth. The main organic products exported are fruits, raisins, olives, and olive oil, while imports focus on processed items like lentils, sugar, cereals, jams, and chocolates [23,28].

Although the organic food market in Greece shows promising growth, it faces hurdles like high prices and supply chain inefficiencies. Organic products are often more expensive due to higher production costs and the lack of large-scale operations. Overcoming these issues will require sustained investment in infrastructure and innovative solutions within the organic agriculture sector [46].

Despite these challenges, there are valuable opportunities for domestic producers and international suppliers to tap into Greece’s increasing demand for organic goods. By forming strategic partnerships, adopting advanced farming technologies, and diversifying their product ranges, stakeholders can better cater to the changing preferences of Greek consumers and drive market expansion [46].

The perception of higher prices and limited availability are widely recognised as the main barriers to the consumption of organic products [8,40,41,47]. Organic products are often perceived as premium items, with prices significantly higher than conventional alternatives [36,48]. Organic products are not as widely available as conventional products, particularly in smaller grocery stores or rural areas. This limited presence makes it harder for consumers to incorporate them into their regular shopping habits. Even in markets with better access, organic sections can be less varied or well-stocked, limiting the options for consumers who prefer organic foods. For routine shopping, consumers often prioritise convenience. If organic products are not readily accessible or require extra effort to source (e.g., visiting speciality stores or farmers’ markets), this can discourage their purchase [36,49]. Availability has consistently been highlighted as a major barrier to purchasing organic products, as when consumers face difficulty in consistently finding organic options in their regular shopping locations, they are less likely to integrate these products into their regular consumption habits. This lack of availability in conventional grocery stores, combined with higher prices, makes organic products less attractive to a broader consumer base [27,40,50,51]. The higher price of organic food has been a significant factor that discourages Greek consumers from purchasing it regularly. In Greece, as in many other countries, the cost of organic products is generally higher than that of conventionally produced goods [28,52]. In addition, the lower availability leads to lower demand, and, as a result, the prices of organic products continue to remain high [52].

The rise in demand for organic products in Greece reflects a broader global trend towards healthier and more sustainable consumption. As consumers increasingly prioritise food quality and environmental impact, understanding the factors influencing their purchasing decisions becomes essential for stakeholders in the organic market. Following similar studies produced previously by the authors, both in Portugal [24] and Spain [26], this study seeks to analyse the behaviours, preferences, and underlying motivations of Greek consumers regarding organic products. The primary objective of this research is to evaluate Greek consumer behaviour concerning organic products. Specifically, we aim to: (i) identify key attributes that influence consumer choice, and (ii) examine the relationship between demographic variables (e.g., age, education, gender) and consumption patterns of organic products.

## 2. Materials and Methods

The questionnaires designed for this study contained informed consent approval before the interviewee started answering the questions. The study was approved by the Department of Animal Science of the University of Thessaly, Greece (reference number 30515/24/TEZP).

As explained, this study is a sister study of previous studies conducted in Portugal and Spain and aims for a comparative study in the future. With this aim in mind, we have standardised methodologies across the studies to allow comparison.

### 2.1. Sampling Method and Questionnaire Design

We have used a quota sampling design in this study, with the values defined proportionally according to the region of residence, age, gender, and academic level. The sample members responded by filling out an online questionnaire. To complete the quotas, face-to-face interviews were carried out. People who consume less than three organic products per week (vegetables, fruits, dairy products, meats, groceries, etc.) were excluded from the research.

This study has an exploratory nature, which, together with the robustness of the BWS, while compared to other methods, allows accurate and credible conclusions [53]. In this study, 250 questionnaires were obtained, with the research taking place between July 2022 and April 2023. Data were collected anonymously, and informed consent was obtained from the interviewees.

Our questionnaire is divided into two parts. There is one with six classification variables: ‘gender’, ‘age’, ‘place of residence’, ‘education level’, ‘children under 18 years old living in the household’, and ‘place of purchase of organic products’. The second part measured the importance of ten organic attributes (‘price’, ‘more natural appearance’, ‘certification warranty’, ‘origin’, ‘expectation of better taste’, ‘availability’, ‘health benefits’, ‘environmental impact’, ‘nutritional value,’ and ‘absence of GMOs’) using the Best-Worst Scaling (BWS) method.

These attributes were chosen based on its importance after the literature review and included ‘health benefits’ [5,24,25,26,54], ‘environmental impact’ [3,9,10,13,34,55], ‘nutritional value’ [15,17,18,38,40], ‘expectation of better taste’ [6,56], ‘price’ [13,47,48,51,54], ‘more natural appearance’ [28,29], ‘certification warranty (EU logo)’ [16,39,40,41,42,44,45,57], ‘’origin [20,35,36], ‘availability’ [27,40,50], and ‘absence of GMOs’ [37].

**Figure 1:**
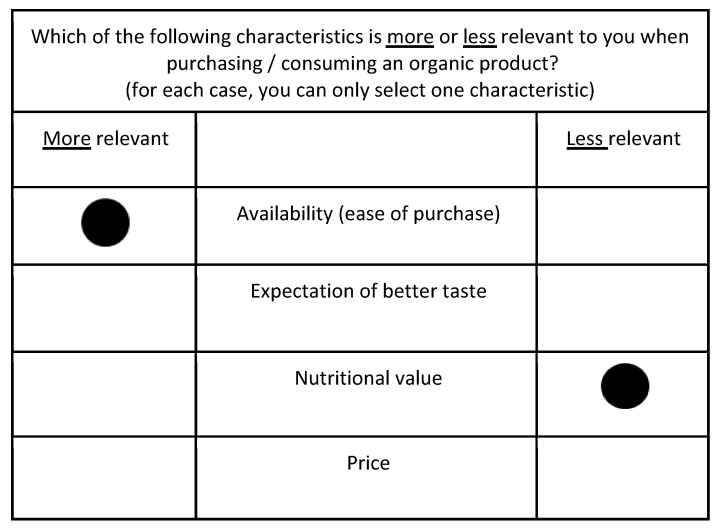
Illustration of the set of choices available in the questionnaire. In the example provided, the interviewee prioritises ‘availability (ease of purchase)’ as the most relevant, while designating the attribute ‘nutritional value’ as the least relevant.

These ten attributes were then combined into a choice of ten sets, each containing four items. There are 210 possible combinations in total, and ten of these possible combinations were randomly made available to the interviewees. Survey interviewees then selected the worst and the best attributes in each of the sets, i.e., the least and most important attributes they consider for the decision of purchasing products of organic origin. Figure 1 below illustrates a choice set after the respondent has submitted their answers. To enhance comprehension of the figure, the text has been translated from Greek to English.

### 2.2. Best-Worst Scaling Methodology

Best-Worst Scaling, also known as Maximum Differentiation (Max-Diff) Scaling, has emerged as a robust survey methodology designed to capture consumer preferences more effectively than traditional rating techniques. Originally conceptualised by Louviere and Woodworth in 1990, BWS addresses several limitations associated with simpler rating scales, such as Likert scales, which can introduce central tendency bias as respondents tend to avoid extreme positions [58].

This innovative approach engages respondents more critically with the items presented to them. It employs a straightforward dual-choice format that enables researchers to identify both the most and least preferred items in each set. The methodology gained initial traction when Finn and Louviere applied it in the context of food safety in 1992. Since then, the versatility of BWS has facilitated its adoption across a wide array of fields. Notably, there has been a significant surge in its application, with approximately 8000 scientific articles featuring BWS published in 2023 and 2024 alone, including around 600 articles focused specifically on food consumer behaviour.

The application of the BWS method in consumer behaviour studies in Greece has been rare, with the work of Chrysochou et al. [59] on cask wine consumption being a notable exception. Given its relatively recent adoption, it is worth highlighting that this study’s methodology, employing maximum differentiation scaling to explore organic consumer behaviour, marks a significant advancement in this type of research within the Greek context.

In employing BWS, researchers begin by identifying a list of relevant attributes for their study—in this study, ten major attributes influencing organic consumption. These attributes are then grouped into choice sets containing typically three to five items, with four items being the most common configuration. Each respondent evaluates several of these sets, generally ranging from eight to ten, allowing for a manageable process that minimises respondent fatigue while ensuring robust data collection. Participants select their best and worst items within each set, and the resulting data are analysed using statistical techniques such as logistic regression, which quantifies the importance of each attribute. For this study, the MaxDiff SSI Web statistical package from Sawtooth Software (Provo, UT, USA) is utilised for both the structuring of the survey and subsequent analysis. The advantages of BWS are compelling. It significantly reduces biases often encountered in traditional survey methodologies, such as central tendency bias and acquiescence bias, while providing clearer insights into consumer preferences. Furthermore, by necessitating prioritisation, BWS fosters a more definitive understanding of the relevance of attributes, thereby informing effective managerial and marketing decisions. In summary, BWS represents a substantial advancement in preference measurement, particularly in its capacity to capture the nuances of consumer behaviour in a format that is less cognitively demanding. This positions BWS as a desirable choice for researchers seeking to deepen their understanding of consumer priorities and develop effective marketing strategies. By leveraging the strengths of BWS, scholars and practitioners can better navigate the complexities of consumer preferences in an increasingly competitive landscape.

Ordinal regressions were used to model the two most important attributes as a function of the classification variables to analyse which of those variables are statistically significant for each of the attributes. Several link functions were tested, and the best fit was chosen. A backward stepwise procedure was then implemented to produce models with only significant variables.

## 3. Results

### 3.1. The Most and the Less Preferred Attributes

According to Loose and Lockshin [60], the frequency with which each attribute is selected as either best (most important) or worst (least important) must be aggregated across all respondents to compute the average best–worst score (B–W)/n for the total sample (see Table 1).

**Figure 2:**
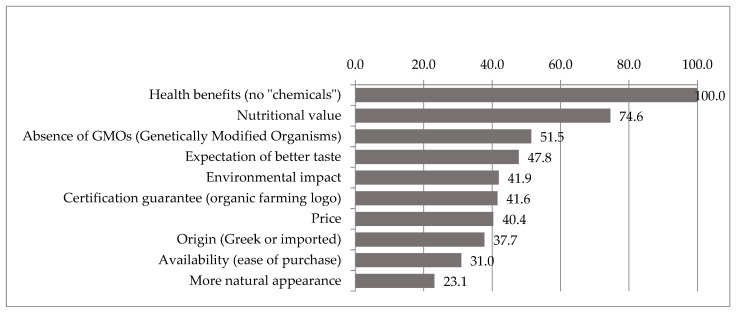
The 10 attributes of organic products displayed on a standardised ratio scale.

This methodology enables researchers to quantify the relative importance of various attributes within the sample population. The correlation between average best-worst scores and those obtained through alternative methods, such as the square-root scale, is often strong; however, it is important to note that these methods may not always produce the same rankings for the assessed attributes. Such discrepancies may arise from differing interpretations of importance and personal preferences as expressed through the distinct selection methods used. In the current study, the attribute ‘expectation of better taste’ illustrates this phenomenon. It ranked third in terms of being selected most frequently as the best attribute, signifying a relatively high perceived importance among respondents. Nonetheless, when analysed using the square root scale, this attribute dropped to the fourth position. This shift in ranking is primarily due to a notable number of respondents who identified it as the least important (worst) attribute, thereby impacting its overall score in the square root scale evaluation. Since the square root scale produces only absolute values, it is essential to calculate a standardised ratio scale index. This allows for a comparative interpretation of all less important attributes as ratios relative to the most important attribute [60]. Sirieix et al. [61] also assert that the standardised ratio scale is a robust index, as it facilitates the interpretation of less relevant aspects as ratios in relation to the most significant attribute. In this context, the attribute with higher significance in our study (‘health benefits’) has been assigned a value of 100, with all other attributes expressed as ratios of this value. To enhance the clarity and visualisation of the data, Figure 2, below, presents the attributes in descending order of relevance based on the standardised ratio scale index.

The attribute ‘health benefits (no chemicals)’ represents the main determinant influencing organic products’ consumption among the studied Greek consumers. This finding aligns with the results of numerous other published studies, where concerns regarding the well-being and health of consumers and their families are foremost among the factors driving organic consumption [24,26,62,63,64,65]. Given that the attribute ‘Health benefits (no chemicals)’ receives a score of 100, it is noteworthy to emphasise that the second most selected attribute possesses a predictive strength of approximately three-quarters that of the first. In other words, although ranked second, the attribute ‘nutritional value’ demonstrates a significant weight in the personal consumption strategies of organic products. Considering previous studies that largely focus on self-centred and selfish attributes, it can be concluded that the relatively high position of this attribute in the current study constitutes a novel aspect worthy of attention. To put it differently, the relatively predictive proximity of the first two selected attributes leaves no room for the recurring assumption that health concerns are, by far, the primary determinants in the decision-making process regarding organic product selection.

The following is an ensemble of five attributes: ‘absence of GMO’, ‘expectation of better taste’, ‘environmental impact’, ‘certification warranty’, and ‘price’. These attributes exhibit a relative mean weight in the consumer behaviour of Greek consumers of organic products, ranging from 51.5 to 40.4, respectively. Following these, the ‘origin’ and ‘availability of organic products’ are ranked as less significant, contributing to approximately one-third of the predictive strength of the most highly selected attribute. Lastly, the ‘perceived naturalness of organic products’ appears to have minimal influence on Greek consumers, as this attribute is ranked lowest among their determinants of organic consumption.

### 3.2. Impact of Classification Variables on Attributes

A chi-square test of independence was applied to identify relationships between classification variables and attributes. However, some cells in the crosstab showed values below one, and more than 20% of these cells had less than five observations, therefore violating the pre-requisites of the test. The problem was solved by merging some of the cells, and all classification variables were transformed to have two classes (Table 2). Regarding places where organic products can be purchased, the ‘generalists’ group refers to places that do not specialise in the sale of organic products; the ‘organic’ group refers to places and/or sales modalities dedicated exclusively (or mainly) to organic products.

Table 3 displays the significance of the relationship between the attributes and the classification variables.

As can be observed, there is no statistically significant relationship between any classification variables and the attributes ‘origin’, ‘expectation of better taste’, and ‘environmental impact’. We have then applied the ordinal regression models to find which independent variables have relevance while explaining each of the attributes.

### 3.3. The Application of the Ordinal Regressions

We started by applying an ordinal regression model to each and all of the dependent variables (attributes) and the classification variables to analyse which of those variables are statistically significant for each of the attributes. The procedure was followed by the detailed analysis of ordinal regressions applied to the two most important attributes where significant relationships with variables were found: ‘health benefits’ and the ‘nutritional value’ (Table 4).

We employed an ordinal regression model for each dependent variable (attribute) in conjunction with the classification variables, aiming to determine which of these variables are statistically significant predictors for each attribute. Subsequently, we conducted a more detailed analysis of the ordinal regressions applied to the two most critical attributes that demonstrated significant relationships with the variables: ‘health benefits’ and ‘nutritional value’ (see Table 4).

An ordinal regression was applied using a complementary ‘log-log link function to find if the independent variables ‘age’, ‘gender’, ‘academic level’, ‘area of residence’, ‘existence of children under 18 years old in the household’, and ‘place to purchase organic food’ show a significant effect on the response probabilities to the attribute ‘health benefits’. The model shows to be statistically significant (χ^2^ = 94.585; 6 df; *p* < 0.001); however, the effect size is not high (R^2^ of Cox and Snell = 0.315). Therefore, we can assume that at least one independent variable contributes with relevance to explaining the mobility in the ‘health benefits’ attribute. The model fits the data (Person’s χ^2^ = 175.039, *p* = 0.079; and deviance = 137.190, *p* = 0.765). The analysis of the significance of the independent variables followed, and the quantification of the parameters associated with the variables found significant was obtained (Table 5). The independent variables found significant were ‘gender’ (Wald χ^2^ = 5.606, *p* = 0.018), ‘academic level’ (Wald χ^2^ = 7.943, *p* = 0.005), ’area of residence’ (Wald χ^2^ = 52.473, *p* < 0.001), and ‘place to purchase organic food’ (Wald χ^2^ = 9.442, *p* = 0.002).

The reduced health benefits attributes model with the significant independent variables only is shown in Table 6. The independent variables found significant were ‘gender’, ‘academic level’, ‘area of residence’, and ‘place to purchase organic food’. The model is statistically significant (χ^2^ = 94.291; 4 df; *p* < 0.001); however, the size of the effect size is not high (R^2^ of Cox and Snell = 0.314). This shows that the four independent variables have a relevant contribution to explaining how the dependent variable varies. The model fits the data (Person’s χ^2^ = 153.989 *p* = 0.084; and deviance = 52.495, *p* = 0.108).

The application of the parallel lines test, confirms the assumption of the slope homogeneity for the model, thus validating it, and as required, slopes homogeneous (Parallel lines χ^2^ = 13.909; 4 df; *p* < 0.084).

According to Table 6, four independent variables (‘gender’, ‘academic level’, ‘area of residence’, and ‘purchasing location for organic food’) significantly contribute to explaining the variation in the dependent variable ‘health benefits’. In terms of gender, the data indicate that male consumers, when compared to the female reference category, demonstrate a lower likelihood of endorsing the higher-order classes of the dependent variable. This finding suggests that female consumers have a greater propensity to acknowledge the health benefits associated with organic products than their male counterparts. Similarly, individuals with higher educational attainment are more likely to recognise the health benefits of organic products than those with lower educational levels.

Regarding the area of residence, the analysis reveals that consumers from Region 1 (Karditsa and Trikala) show a higher likelihood of agreeing with the health benefits of organic products compared to consumers in Region 2 (Larissa, Magnesia, and Sporades). This indicates that residents of Region 1 are more supportive of the health benefits associated with organic offerings. Additionally, consumers who purchase organic products from general supermarkets or hypermarkets tend to value the health benefits of these products more than those who exclusively seek out specialised organic stores.

### 3.4. The Attributes Related to Nutritional Value

An ordinal regression was applied using a negative log-log link function to assess whether ‘age’, ‘gender’, ‘academic level’, ‘area of residence’, ‘existence of children under 18 years old in the household’, and ‘place to purchase organic food’ have a statistically significant effect on the response probabilities to the attribute ‘nutritional values’. The model was found to be statistically significant (χ^2^ = 73.080; 6 df; *p* < 0.001), even though the size of the effect is somewhat reduced (R^2^ of Cox and Snell = 0.253). Therefore, we can assume that at least one of the independent variables has a relevant contribution to explaining the variation in the attribute ‘nutritional value’. The model fits the data (Person’s χ^2^ = 158.250, *p* = 0.306; and deviance = 145.748, *p* = 0.583). The analysis of the significance of the independent variables followed, and the quantification of the parameters associated with the variables found significant was obtained (Table 7). The independent variables found significant were ‘academic level’ (Wald χ^2^ = 8.594, *p* = 0.018), ‘area of residence’ (Wald χ^2^ = 53.444, *p* < 0.001) and ‘place to purchase organic food’ (Wald χ^2^ = 4.376, *p* = 0.036).

The reduced nutritional value attributes model with the significant independent variables only is shown in Table 8. The independent variables found significant were ‘academic level’, ‘area of residence’, and ‘place to purchase organic food’. The model is statistically significant (χ^2^ = 70.885; 43 df; *p* < 0.001); however, the effect size is not high (R^2^ of Cox and Snell = 0.247). This highlights that the four independent variables contribute in a relevant way to explain the variation in the dependent variable. The model fits the data (Person’s χ^2^ = 26.623 *p* = 0.086; and deviance = 23.358, *p* = 0.177).

The application of the test of parallel lines test, the assumption of the slope homogeneity for the model was validated, and as required, slopes are thus homogeneous (Parallel lines χ^2^ = 11.617; 3 df; *p* < 0.477).

According to Table 8, three of the independent variables (‘academic level’, ‘area of residence’, and ‘place to purchase organic food’), contribute in a relevant way to explain the variation in the dependent variable of nutritional value. Concerning the academic level, we can observe that individuals without higher education and in relation to those with higher education are less likely to buy organic products. Consumers with higher education backgrounds are more in agreement with the nutritional value of organic products than consumers without higher education background.

Regarding the area of residence, it is observed that for Region 1 relative to Region 2, the lower-order classes of the dependent variable are less likely than higher-order classes to buy organic products. Therefore, consumers from the regions of Karditsa and Tricala agree more with the nutritional value of organic products than consumers from Larissa, Magnesia, and Sporades, as a whole. In the same way, consumers buying organic products in undifferentiated stores, such as hypermarkets and supermarkets, consider the nutritional value more than consumers buying in specialised organic stores.

## 4. Discussion

### 4.1. The Importance of the Different Organic Food Attributes Among the Greek Population Studied

While studying the importance given to the different attributes of organic food, we found that the most significant attribute is ‘health benefits (no chemicals)’. Other important attributes are (by decreasing order of importance) ‘nutritional value’, ‘absence of GMOs’, ‘expectation of a better taste’, and ‘environmental impact’. The attributes with lower importance are (by decreasing order) ‘certification’, ‘price’, ‘country of origin’, ‘availability’, and ‘more natural appearance’.

The health benefits of organic food consumption are increasingly supported by scientific evidence. Jiang et al. [66] produced a systematic review on the topic revealing that organic diets significantly lower pesticide residue intake, which may reduce risks associated with long-term exposure, such as developmental or neurological effects. The same study also links organic food consumption with lower risks of obesity, diabetes, and cardiovascular issues, likely due to fewer contaminants and higher bioactive compounds, such as antioxidants. The prominence of health benefits reflects a widespread perception that organic food is safer due to the absence of synthetic pesticides and fertilisers in the Greek population [67]. This aligns with research showing that consumers are primarily motivated by the desire to avoid chemical residues in food. Health consciousness is one of the most significant drivers of organic food consumption [31,68]. Organic food is often marketed with claims of reduced pesticide exposure. The prioritisation of health as the main attribute of organic food was also previously identified in the Greek population by [23,27], which identified that Greek consumers consider food safety as a main attribute of organic food.

The fact that organic food offers superior nutritional value has been a contested area in research. While some meta-analyses, such as the one by Barański et al. [69], indicate higher antioxidant levels in organic produce, others suggest that nutritional differences between conventional and organic products are minimal [18]. More recently, a comprehensive analysis noted that in addition to antioxidants, organic foods often contain higher levels of iron, magnesium, and vitamin C [70]. A study conducted on Greek products [71] has shown minor evidence of an association between organic food and higher nutritional content. Nevertheless, a large majority of consumers tend to perceive organic food as being more nutritious [72].

The absence of genetically modified organisms (GMOs) in organic food has also been identified as a critical factor for consumers, particularly in Europe, where public opposition to GMOs is high [73]. In the EU, organic certification prohibits GMOs (EU regulation 848/2018), assuring consumers who are wary of potential risks associated with genetic modification. This sentiment is reflected in broader trends across the EU, where GMO-free labelling significantly influences purchasing behaviour [74]. The EU, as opposed to the USA, applies the precautionary principle concerning food products, and this principle is rooted in European consumer behaviour [75]. Greek consumers’ preference for organic food to avoid GMOs was highlighted by Diagourtas et al. [27] and by Katidi et al. [71].

Taste is a subjective but essential factor driving organic food consumption. Studies like that of Nadricka et al. [20], reveal that consumers perceive organic products as tastier and more natural, even when blind taste tests do not always confirm these differences. This expectation may be influenced by a psychological bias known as the “halo effect”, where organic labelling enhances the perceived quality of food [76]. This “halo effect” persists also with the healthier and more nutritious perception towards organic food [77]. Nevertheless, consumers in Greece have listed the taste of traditional food as a main attribute at the time of purchasing food [78].

The environmental benefits are an increasingly recognised attribute of organic farming, which uses practices that reduce pollution, conserve water, and promote biodiversity [79]. The prioritisation of this attribute suggests an awareness of the environmental footprint of food production, reflecting global trends toward sustainable consumption [23,80]. In a previous study conducted by Malissiova and colleagues., the Greek consumer did not identify environmental protection as a main attribute of organic food [23]. Nevertheless, organic food choice has been identified as an important attribute at the time of choosing organic food among Greeks worried about climate change [81].

Certification ranks below the above attributes, likely because consumers assume certified products inherently meet their expectations for organic standards. However, studies like Murphy et al. [82] emphasise that certification schemes play a crucial role in building trust, especially in markets where organic fraud has occurred. In a survey conducted by Eurobarometer [83] in 2022, Greeks show giving lower levels of importance to certification of food in comparison to other EU countries.

Although price is a critical barrier to organic food consumption globally [49,84], its relatively lower importance in this context suggests that Greek consumers prioritise health and sustainability over cost. However, affordability remains a challenge and has been identified as a barrier to organic food consumption by the Greeks [23].

The country of origin’s lower importance does not reflect the growing interest in supporting local production and reducing food miles. Greek consumers prefer domestic products [78]; however, in the present study, this is not one of the main attributes selected as the most important in buying organic food.

Availability often ranks low in organic food attribute importance because it is a practical consideration rather than an intrinsic product attribute. However, studies such as Tarkiainen and Sundqvist [85] found that consumers may be discouraged from purchasing organic food products when availability is restricted. Conversely, when organic options are readily accessible, this convenience can enhance consumer interest and increase purchase rates.

The lower importance given to appearance indicates that Greek consumers prioritise intrinsic qualities like health and environmental impact over extrinsic ones. However, natural appearance remains an attribute for some, as it signifies authenticity and reduced artificial handling, as suggested by Eyinade et al. [86].

### 4.2. Identification of Health and Nutritional Cross-Benefits of Organic Food Consumption: Demographics, Type of Store, and Regional Variability

In our study, Greek women have shown a greater propensity to acknowledge the health benefits of organic food in comparison with men. Women often exhibit greater health consciousness compared to men and are more likely to engage with health-related messaging and prioritise natural food choices. Women’s roles in household food provisioning may also contribute to their heightened interest in health, and nutrition-related food attributes. Female consumers tend to care more about health benefits [87,88,89], natural food [89], ecolabels [90], and sustainability [91]. Overall, women have a higher propensity to act as active food consumers, taking time to look for better choices, while men act as passive consumers [92]. In a study conducted in Greece, Malissiova et al. [23] found that Greek women are more aware of organic products and show a higher propensity for consumption, which tallies with our results.

Similarly, individuals with higher educational attainment are more likely to recognise the health benefits associated with organic food than those with lower educational levels. Higher education levels are strongly associated with increased awareness of the health and environmental impacts of food production [93]. Educated individuals are more likely to access and understand scientific information about the benefits of organic products, as noted by Mata and Dos-Santos [94]. Education influences critical thinking, which helps consumers evaluate the authenticity of health claims associated with organic labelling [95]. In Greece, a previous study [23] showed that higher levels of education correlate positively with a higher propensity for organic food consumption.

Concerning the location of organic food purchasing, our study suggests that in Greece, consumers purchasing organic products in generic super and hypermarkets tend to value the health benefits of these products, in comparison to consumers purchasing in specialised organic stores. According to Malissiova et al. [23], supermarkets are identified by the Greeks as having the best prices and the easiest access to organic food, while speciality stores have a greater variety of products and better service. According to Bimbo et al. [96], people who are environmentally conscious and actively engage in sustainable practices are more inclined to purchase locally sourced food. The same authors report that regular habits like reading nutrition labels, residing in smaller communities, and choosing organic products also serve as strong indicators of a greater preference for local food options.

The distribution of organic food products in Greece goes exclusively through general retailers (supermarkets) and specialised organic products stores. In the 2000s, specialised organic stores were the basic market channel through which organic food products would end up in the household basket. Since 2010, supermarkets have become the driving force in the Greek organic food market, while specialised retailers are facing more competition. Organic food consumption in Greece remains extremely low compared to other countries with a similar share of organic crop area [27].

Residents in the regions of Karditsa and Trikala (Region 1) are more supportive of the health benefits associated with organic offerings, in comparison to those of Larissa, Magnesia, and Sporades (Region 2). The more positive perception of organic product nutritional value among consumers in Region 1 can be ascribed to several significant factors: (i) Closeness to rural lifestyles—Region 1 is rural with stronger ties to farming, enhancing residents’ understanding and trust in organic foods. In contrast, urban areas in Region 2 are more distanced from agricultural practices; (ii) Cultural traditions—The traditional diets of Region 1 prioritise fresh, local foods, making residents more aware of the nutritional benefits of organic products. In urban and tourism-oriented regions, convenience foods may lead to less focus on these distinctions; (iii) Economic access—Consumers in Region 1 often have better access to affordable, locally produced organic goods, reinforcing their perceived value. In urban areas, like Region 2, organic products may be viewed as premium items, leading to some scepticism about their nutritional benefits, and (iv) Trust in sourcing—Residents of Region 1 tend to trust local farmers and their organic claims, while urban consumers may be more sceptical of commercially labelled organic products, viewing them as potential marketing strategies rather than genuine improvements.

### 4.3. Limitations, Future Paths for Research, and Recommendations for Stakeholders

The present study has limitations and offers avenues for future research. It is constrained by its geographic focus, limiting generalizability to the entire country. Also, its cross-sectional format was designed to capture a screenshot of the moment, which does not capture changing consumer trends over time. Self-reported data may be affected by biases, and the study lacks differentiation across organic product categories.

Future research should conduct cross-country studies to identify main market tendencies, instead of local and national trends. The use of longitudinal methods can be used to track evolving preferences, and segmentation analyses can be used to understand subgroup priorities. Behavioural analytics can complement self-reported data while exploring the effects of policy changes. This would address gaps in consumer behaviour research and guide targeted strategies in organic food marketing.

To boost organic food adoption, stakeholders should emphasise the health benefits of organic products, as this is the primary concern for Greek consumers. Enhanced marketing strategies should highlight reduced chemical exposure, nutritional advantages, and the absence of GMOs. Investment in education and awareness campaigns, particularly targeting women and highly educated demographics, can improve understanding of organic certifications and their credibility. Expanding availability through supermarkets and offering competitive pricing can attract more consumers. Partnerships with local farmers and promoting regional products can build trust and improve access. Lastly, adopting innovative farming methods and improving supply chain efficiency will help overcome cost and distribution challenges.

## 5. Conclusions

The study on Greek consumers’ perceptions of the attributes of organic products reveals critical insights into consumer priorities and behaviours. Health benefits emerged as the most significant attribute, related to scientific evidence suggesting that organic food has reduced pesticide exposure and associated health risks. Nutritional value, absence of GMOs, and taste were also highly valued, reflecting widespread perceptions that organic food offers superior quality and safety. The environmental impact was also considered important among certain groups, mainly women and higher-educated individuals, highlighting growing awareness for sustainable practices in food production. Country of origin, price, availability, and appearance were less influential attributes, suggesting that Greek consumers prioritise intrinsic qualities over practical or aesthetic aspects. Women and individuals with higher education levels were more likely to recognise the health benefits of organic food, possibly due to greater health consciousness and access to information.

These findings are consistent with broader European consumer trends and emphasise the need for organic producers to focus on health claims and sustainability messaging to align with consumer values. Supermarket shoppers valued health benefits more than those frequenting specialised organic stores, likely influenced by marketing strategies and product accessibility. These results provide insights into the market segmentation of organic food consumers, and tailored marketing strategies may be crucial to capturinconsumers’ attention: supermarkets could emphasise health benefits to attract and retain health-conscious customers, while specialised stores might benefit from highlighting sustainability or local sourcing. Regional differences are noticed, with rural residents displaying stronger support for organic food benefits, likely due to closer ties to agriculture and cultural traditions.

## Figures and Tables

**Table 1:** Raw best and worst, average best-worst, and standardised aggregated importance weights.

Attribute	IN	TSB	TSW	(B-W)/n	Sqrt(B/W)	SRS	SIW (%)
Health benefits (no “chemicals”)	7	450	94	1.424	2.188	100	20.43
Nutritional value	9	298	112	0.744	1.631	74.6	15.23
Absence of GMOs (Genetically Modified Organisms)	10	199	157	0.168	1.126	51.5	10.51
Expectation of better taste	5	200	183	0.068	1.045	47.8	9.76
Environmental impact	8	133	158	−0.1	0.917	41.9	8.57
Certification guarantee (organic farming logo)	3	172	208	−0.144	0.909	41.6	8.49
Price	1	170	218	−0.192	0.883	40.4	8.25
Origin (Greek or imported)	4	156	229	−0.292	0.825	37.7	7.71
Availability (ease of purchase)	6	129	281	−0.608	0.678	31	6.33
More natural appearance	2	92	359	−1.068	0.506	23.1	4.73

Note: IN—Item number, TSB—Times selected best, TSW—Times selected worst, SRS—Standardised ratio scale, SIW—standardised importance weights.

**Table 2:** Structure of the sample.

Ner	Classification Variable	Modality	n	
1	Age	≤54 years old	159	250
		≥55 years old	91	
2	Gender	Male	115	250
		Female	135	
3	Academic level	Not superior	215	250
		Superior (degree or more)	35	
4	Area of residence	Region 1 = Karditsa, Trikala	79	250
		Region 2 = Larissa, Magnesia, Sporades	171	
5	Do you have children under 18 living in your household?	Yes	109	250
		No	141	
6	What is the best place to purchase certified organic products?	Generalist = fairs/producer markets, general super and hypermarkets (no certified organic)	133	250
		Organic = organic supermarkets, home delivery baskets, natural/local stores (mostly certified organic)	117	

**Table 3:** Chi-square test for independence (*p*-values). Cells marked with (a) in superscript have significant relationships (*p* < 0.05) and cells marked with (b) in superscript have a tendency for relationships (0.05 < *p* < 0.1).

	Classification Variable					
Attribute	Age	Gender	AL	AR	C18	PPOF
Price	0.712	0.434	0.166	<0.001 ^a^	0.797	0.455
More natural appearance	0.166	0.034 ^a^	0.014 ^a^	<0.001 ^a^	0.829	0.804
Certification warranty (EU logo)	0.492	0.538	0.305	<0.001 ^a^	0.566	0.474
Origin	0.208	0.978	0.065 ^b^	0.297	0.386	0.699
Expectation of better taste	0.318	0.490	0.913	0.574	0.132	0.307
Availability	0.983	0.017 ^a^	0.151	<0.001 ^a^	0.148	0.990
Health benefits	0.864	0.140	0.008 ^a^	<0.001 ^a^	0.283	0.339
Environmental impact	0.511	0.666	0.189	0.095 ^b^	0.104	0.161
Nutritional value	0.645	0.417	0.235	<0.001 ^a^	0.381	0.629
Absence of GMOs	0.885	0.005 ^a^	0.107	0.002 ^a^	0.492	0.384

Note: AL—Academic level; AR—Area of residence; C18—Children under 18 years old in the household; PPOF—Place of purchase of organic food.

**Table 4:** The models of ordinal regression on the attributes and classification variables.

Attribute		Link Function	-2LL Sig.	Pearson Sig.	Parallel Sig.	Significance Levels in Independent Variables						-2LL	LI
						Age	Gender	AL	AR	C18	PPOF		
1	Price	1	<0.001	0.998	0.175	----	----	----	<0.001 ^b^	----	----	<0.001	7
2	More natural appearance	1	<0.001	0.213	0.447	----	0.021 ^a^	0.001 ^a^	<0.001 ^b^	----	----	<0.001	10
3	Certification warranty (EU logo)	1	<0.001	0.615	0.144	----	----	----	<0.001 ^b^	----	----	<0.001	6
4	Origin	1	0.071	0.579	----	----	----	----	----	----	----	----	8
5	Expectation of better taste	1	0.343	0.446	----	----	----	----	----	----	----	----	4
6	Availability	1	<0.001	0.039	0.203	----	0.009 ^a^	----	<0.001 ^b^	----	----	<0.001	9
7	Health benefits	2	<0.001	0.079	0.084	----	0.019 ^b^	0.005 ^b^	<0.001 ^a^	----	0.002 ^a^	<0.001	1
8	Environmental impact	1	0.053	0.989	----	----	----	----	----	----	----	----	5
9	Nutritional value	2	<0.001	0.306	0.440	----	----	0.004 ^b^	<0.001 ^a^	----	0.042 ^a^	<0.001	2
10	Absence of GMOs	1	0.001	0.084	0.357	----	----	----	<0.001 ^a^	----	----	<0.001	3

Note: AL—Academic level, AR—Area of residence, C18—Children under 18 years old in the household, PPOF—Place of purchase of organic food, LI—Level of importance, LL—Log likelihood; Link Function: 1—Negative Log-log; 2—Complementary Log-log; Significance Levels: (^a^)—The variable-ratio is positive; (^b^)—The variable-ratio is negative. The attributes related to health benefits.

**Table 5:** Parameterisation of the health benefits attributes model with all the independent variables.

Variable		*β*	SE	Wald χ^2^	df	*p*-Value	95% Confidence Interval	
							LB	UB
Threshold	HB = 1	−2.032	0.375	29.379	1	<0.001	−2.767	−1.297
HB = 2		−0.986	0.355	7.691	1	0.006	−1.682	−0.289
HB = 3		−0.356	0.349	1.043	1	>0.05	−1.040	0.327
Age	≤54	−0.062	0.181	0.117	1	>0.05	−0.418	0.294
≥55		Reference						
Gender	Male	−0.415	0.175	5.606	1	0.018	−0.758	−0.071
Female		Reference						
Academic Level	NHE	−0.812	0.288	7.943	1	0.005	−1.377	−0.247
HE		Reference						
Residence	Region 1	2.015	0.278	52.473	1	<0.001	1.470	2.560
Region 2		Reference						
Children U18	Yes	−0.083	0.174	0.226	1	0.634	−0.424	0.258
No		Reference						
PPOF	Generalist	0.545	0.177	9.442	1	0.002	0.197	0.893
Organic		Reference						

Note: SE—Standard Error, df—degrees of freedom, LB—Lower bound, UP—Upper bound, HB—Health benefit, NHE—Non higher education, HE—Higher education, U18—Under 18 years old, PPOF—Place of purchase of organic food.

**Table 6:** Parameterization of the health benefits attributes reduced model with the significant independent variables only.

Variable		*β*	SE	Wald χ^2^	df	*p*-Value	95% Confidence Interval	
							LB	UB
Threshold	HB = 1	−1.929	0.329	34.456	1	<0.001	−2.573	−1.285
HB = 2		−0.883	0.307	8.261	1	0.004	−1.485	−0.281
HB = 3		−0.256	00.300	0.728	1	0.394	−0.845	0.332
Gender	Male	−0.410	0.175	5.505	1	0.019	−0.753	−0.068
Female		Reference						
Academic Level	NHE	−0.806	0.288	7.840	1	0.005	−1.371	−0.242
HE		Reference						
Residence	Region 1	2.017	0.278	52.682	1	<0.001	1.472	2.562
Region 2		Reference						
PPOF	Generalist	0.556	0.176	9.943	1	0.002	0.210	0.901
Organic		Reference						

Note: SE—Standard Error, df—degrees of freedom, LB—Lower bound, UP—Upper bound, HB—Health benefit, NHE—Non higher education, HE—Higher education, PPOF—Place of purchase of organic food.

**Table 7:** Parameterisation of the nutritional value attributes model with all the independent variables.

Variable		*β*	SE	Wald χ^2^	df	*p*-Value	95% Confidence Interval	
							LB	UB
Threshold	HB = 1	−1.828	0.326	31.369	1	<0.001	−2.467	−1.188
HB = 2		−0.698	0.307	5.179	1	0.023	−1.300	−0.097
HB = 3		0.182	0.300	0.369	1	0.544	−0.406	0.771
Age	≤54	−0.096	0.161	0.358	1	0.549	−0.411	0.219
≥55		Reference						
Gender	Male	−0.206	0.154	1.796	1	0.180	−0.507	0.095
Female		Reference						
Academic Level	NHE	−0.699	0.238	8.594	1	0.003	−1.166	−0.232
HE		Reference						
Residence	Region 1	1.445	0.198	53.444	1	0.000	1.058	1.832
Region 2		Reference						
Children U18	Yes	−0.091	0.155	0.346	1	0.556	−0.394	0.212
No		Reference						
PPOF	Generalist	0.327	0.156	4.376	1	0.036	0.021	0.633
Organic		Reference						

Note: SE—Standard Error, df—degrees of freedom, LB—Lower bound, UP—Upper bound, HB—Health benefit, NHE—Non higher education, HE—Higher education, U18—Under 18 years old, PPOF—Place of purchase of organic food.

**Table 8:** The parameterisation of the nutritional value attributes reduced model with the significant independent variables only.

Variable		*β*	SE	Wald χ^2^	df	*p*-Value	95% Confidence Interval	
							LB	UB
Threshold	HB = 1	−1.614	0.272	35.198	1	<0.001	−2.147	−1.080
HB = 2		−0.490	0.250	3.836	1	0.050	−0.980	0.000
HB = 3		0.388	0.245	2.513	1	0.113	−0.092	0.867
Academic Level	NHE	−0.688	0.238	8.384	1	0.004	−1.153	−0.222
HE		Reference						
Residence	Region 1	1.444	0.197	53.715	1	<0.001	1.058	1.831
Region 2		Reference						
PPOF	Generalist	0.311	0.153	4.123	1	0.042	0.011	0.610
Organic		Reference						

Note: SE—Standard Error, df—degrees of freedom, LB—Lower bound, UP—Upper bound, HB—Health benefit, NHE—Non higher education, HE—Higher education, PPOF—Place of purchase of organic food.

## Data Availability

The data used in this study are available upon reasonable request from the corresponding author only due to confidentiality restrictions.
